# Smells, and Their Management

**Published:** 1862-04

**Authors:** Geo. Watt


### DENTAL REGISTER OF THE WEST.

Vol. XVI.] APRIL, 1862. [No. 4.

### Original Essays and Communications.

### SMELLS, AND THEIR MANAGEMENT.

BY DR. GEO. WATT.

When a certain Spartan woman visited a lady friend, we are told by Plutarch that one of them was so thoroughly perfumed with "sweet ointment," and the other with *butter*, that they were not able to endure each other's presence. The *sweet* ointment is of doubtful composition; but she that objected to the butter had substantial reasons for her disgust, as, when used as an ointment, on account of the great extent of surface exposed, it would decompose rapidly, and, by the formation of butyric acid, a genuine *stink* would be produced.

In these days of perfumes and cosmetics, there is at least some danger that similar mutual disgust may be engendered between the dentist and his patients. True, the dentist, for sake of patronage, is expected to endure the hardship of stinks without a murmur; and in this manner he should endure all that are unavoidable. But when a little common sense and practical knowledge will do much to overcome such difficulties, justice to the olfactory nerves requires a proper application of this sense and knowledge. And a decent regard for the comfort of his patients, in connection with a due appreciation of his own interests, would lead him to give close attention to the prevention of unpleasant odors, both about his person and office. And, as tastes are so varied that it is impossible to tell what odors any given individual would enjoy, good sense would teach him, when at all possible, to guard against the presence of any odor whatever. *Odorless perfumes*, in the language of the "green isle," will meet with the fewest objectors.

While it is true that people differ widely in regard to their choice of odors, yet there are some smells that are almost unanimously regarded as unpleasant. Some of these are natural productions; others are formed by art. A few only will be noticed in this connection.

HYDROSULPHURIC ACID.—This acid, often called *sulphuretted* hydrogen, occurs abundantly in nature, and is readily formed by art. If iron filings and sulphur are heated together in a crucible, in the proportion of 28 parts of iron to 16 of sulphur, they combine forming a black substance called *sulphuret of iron.* When sulphuric acid is poured on this, a gas is given off, having a strong offensive smell like that of rotten eggs. This gas is not only offensive, but highly poisonous when inspired. Water dissolves about two and a half times its bulk of it, which is again expelled when the water is boiled.

A person who indulges in the excessive use of albuminous food, or otherwise introduces sulphur into his system, exhales this acid both by expiration and through the pores of the skin. Every dentist who has an experience at the chair has found patients whose breaths were loaded with this fetid gas; and he has found himself far more relaxed and exhausted after operating for such a patient, than after waiting on one whose breath was normal. It is not often practicable to rectify the patient's breath before operating, though in a few cases we do it; but, from the effects of the patient's breath on himself, the dentist should learn a lesson in regard to the possible effect of his on the patient. And a little carelessness in this respect may lose him the patronage of many valuable, though sensitive, patients.

This gas is also formed in the mouth when albuminous matters are allowed to putrefy, or decompose in it; and, being soluble in water, the saliva, from this source, as well as from the breath, is often found highly impregnated with it. Its oxydation here is the probable origin of the sulphuric acid which produces the black variety of dental caries. When the saliva contains it, it throws down a black precipitate (*sulphuret of lead*) from a solution of acetate of lead. This test is sufficiently reliable for the dental office.

This gas is a principal cause of the inordinate stench arising from the putrefaction of dead carcasses, or of animal matters generally. Hence, the dentist needs not be told that if *blood* be allowed to decompose in, or about his spittoon, he must expect to meet with it. And here it may be well enough to remark that too great care can not be exercised, either about the person or office of the operator; for a smell that would be highly offensive to a sensitive patient might not be observed at all by him, as by its gradual manifestation his senses may have been taught to tolerate it.

PHOSPHURETTED HYDROGEN is another offensive gas formed in the putrefaction of animal matter; and many of the general remarks on the sulphuretted hydrogen are equally applicable to this.

AMMONIA is also formed and given off under similar circumstances; and, commingled with the two gases above considered, forms one of the most loathsome stenches known in nature.

The sweat contains a number of odorous principles, none of them very agreeable under the circumstances in which they are presented, and some of them very highly offensive. Many strongly scented substances, when taken into the stomach, pass into the circulation and are exhaled with the perspiration. The odorous principles of garlic, onions, tobacco, etc., are familiar illustrations of this fact. But offensive odors seem to pertain to normal sweat, their quantity and offensiveness varying greatly in different persons.

These odorous principles are not all thoroughly understood. It is pretty well established that butyric acid is ordinarily present, which is sufficiently offensive to disgust even those who are not fastidious. But this does not explain all; for the perspiration of the feet, axillæ, and groins is often, indeed generally, more fetid than that of other parts, while it usually gives an alkaline reaction.

The eating of onions and garlic, and the chewing or smoking of tobacco are often the means of introducing the most offensive smells into the dental office. When introduced by the patient, he should be charged double price for each operation performed,—when by the operator, the patient should leave the office without hesitation or ceremony. It is no mitigation that ladies often politely *pretend* that these smells are not offensive. It is to be hoped that a kind Savior will pardon their goodnatured hypocrisy, and that his Holy Spirit will purify their hearts from it.

A frequent cause of a fetid breath is a morbid secretion of the tonsils and the parts in their vicinity. Of the composition of the odorous principle of this secretion we know but little. The sebacious secretions, too, often become very offensive when cleanliness is neglected.

But we have no more space to devote to smells. The question arises as to their management. The agencies employed in this direction may be divided into *smell-preventers*, *smell-disguisers*, *smell-removers*, and *smell destroyers*. Or they may be called *antiseptics*, *perfumes*, *deodorizers*, and *disinfectants*.

The ordinary processes of preserving meat are familiar proofs that offensive smells may be prevented. But about the dental office, substances likely to produce them should be carefully removed before they have time to decompose. It is not always practicable to have the spittoon so perfectly arranged that all traces of blood are instantly washed into a sink by a current of water. Hence the necessity of preventing its putrefaction, which, in hot weather, is very rapid.

With the most careful removal, slight traces of blood often remain, and prove highly offensive to sensitive patients. The stench, from this source, is mostly due to sulphuretted and phosphuretted hydrogen, and ammonia. And their formation may be mainly or wholly prevented by the presence, in contact with the blood, of common salt, sugar, quicklime, nitrate of potash, sulphate of iron, or vegetable astringents.

But when the unpleasant odors are once formed, let them be *removed* or *destroyed.* They should never be *disguised.* It has been well remarked by Johnson that when we mingle with the smell which we dislike some odor we can enjoy, we "leave floating in the air around us the evil and the good together, to produce unheeded their natural effects upon the system." He says, further:—"Sweet odors are thus the natural disguises of evil smells. They are the only resource of rude and dirty times against the offensive emanations from decaying animal and vegetable substances, from undrained and untidy dwellings, from unclean clothes, from ill-washed skins, and from ill-used stomachs. The scented handkerchief in these circumstances takes the place of the sponge and shower-bath; the pastile hides the want of ventilation; the ottar of roses seems to render the scavenger unnecessary, and a sprinkling of musk sets all other stinks and smells at defiance."

Substances used for the *removal* of smells are called deodorizers; and it should be always remembered that cleanliness and ventilation are generally the great agencies, and though they may sometimes fail to accomplish all that is desirable, yet they are never inappropriate.

Charcoal has long held a prominent place among the deodorizers. Like other porous bodies it concentrates gases and vapors in its pores. From its great porosity, and from the fact that it suffers no chemical change in the process, it is very efficient in this respect. Freshly calcined, light wood-charcoal absorbs nearly 100 times its bulk of ammonia,

50 times its bulk of hydrosulphuric acid, nearly 10 of oxygen, and varying proportions of other gases.

While this condensing power of charcoal is due mainly, if not solely, to its *physical* property of porosity, yet active chemical changes are induced by it. For example, when various gases are thus condensed, they are brought within the range of their affinities—are more *intimately in contact* than in their ordinary diffused state. When sulphuretted hydrogen and oxygen (from the atmosphere) are thus in contact, the sulphur and hydrogen are both oxydized, forming sulphuric acid and water, as represented in the following formula:

$$HS + O_4 = HO + SO_3.$$

Phosphuretted hydrogen is similarly oxydized under like circumstances, phosphoric acid and water being the results. Ammonia also suffers a like fate by the formation of *nitric acid* and water, the reaction being indicated thus:

$$NH_3 + O_8 = 3\,HO + NO_5.$$

The non-chemical reader will please understand that ammonia is composed of nitrogen and hydrogen, as represented by the symbols, $NH_3$.

Calcined clay, the ashes of bituminous coal, coke, and many other porous substances, have the same effect as charcoal, but in a less degree. But with the exception of what can be accomplished by cleanliness and ventilation, smell-removing is not as well adapted to the wants of the dental office as smell-destroying.

There is a radical and highly practical difference between *deodorizers* and *disinfectants*—between smell-removers and smell-destroyers. Water, as already referred to, may remove sulphuretted hydrogen from the atmosphere by dissolving it, but on the application of heat, or the gradual evaporation of the water, the gas escapes unchanged. But far different is the case when this, or a similar gas, is acted on by a *disinfectant.* The disinfectant either combines with it, forming a non-offensive compound; or it decomposes it.

It is not our purpose to notice all, or even many of the substances relied on as disinfectants. A proper understanding of the few most convenient, and most applicable to the wants of the dental office, is far more desirable than indefinite ideas extended over a much wider range.

*Sulphurous acid* is a cheap and powerful disinfectant, but has many objectionable properties which nearly exclude it from the dental office. It is readily and rapidly formed by burning sulphur, or by immersing copper in sulphuric acid. By virtue of its acidity, it neutralizes alkaline substances, and of course, therefore, ammonia, one of the offensive results of animal putrefaction. Its great affinity for oxygen enables it to decompose most odorous compounds containing that element. But being itself highly offensive to the smell, and almost irrespirable, added to the fact that instruments of steel are rapidly corroded when exposed to its action, it is not adapted to the dental office, as such, but is very effectual, cheap, and convenient, in purifying houses which have been neglected, provided the process is completed and the house ventilated before occupying it.

*Chlorine*, however, is quite as reliable, and in many cases more so than the sulphurous acid. It, too, indirectly, corrodes our instruments if left to come in contact with it; and it is a *very* suffocating gas, but is more manageable than that just considered. It may be obtained very cheaply by mixing together common salt and black oxyd of manganese, and pouring sulphuric acid (oil of vitriol) over them. As all the materials are cheap, minute attention to proportions is not necessary. The action, too, is more satisfactory when the acid is somewhat diluted.

Chlorine is not only the great *bleaching agent*, but the great disinfectant. By virtue of its affinity for hydrogen, it readily decomposes sulphuretted hydrogen, phosphuretted hydrogen, and ammonia, as well as nearly all other odors arising from the putrefactive process. In the presence of moisture, it takes hydrogen from the water, leaving the oxygen, in its nascent state, to act with an energy but little, if at all, inferior to its own. Indirectly, it is thus the great *oxydizing* agent.

Chlorine is very efficient as a disinfectant, even when greatly diluted with atmospheric air, and vapor of water. When thus highly diluted it may be breathed without either difficulty or danger, and sometimes, as in putrescent diseases, with advantage.

The *chloride of lime*, of commerce, which is the hypochlorite of lime mixed with hydrate of lime, is more convenient in many cases than chlorine. When a solid or liquid disinfectant is desired, this is at once suggested. Its action is not as unlike that of chlorine as some have maintained. The lime which it contains, by its alkaline properties, can neutralize, at least to a good degree, acid odors; but the hypochlorous acid is the great disinfectant principle of this compound. This acid, as stated in a former article, (see "Calcium and its Compounds,") is not a permanent compound, but is readily decomposed; and thus the chlorine and oxygen, of which it is composed, are both presented in their nascent state, to act on the odorous compound, whatever it may be.

Let us suppose, then, that this substance (the chloride of lime) is brought in contact with blood, or other animal matter in the process of putrefaction, and consider the chemical actions which result.

Ammonia, phosphuretted hydrogen, and sulphuretted hydrogen all contain hydrogen as one of their constituent elements. Hypochlorous acid is composed of one equivalent of chlorine, and one of oxygen. This acid will be rapidly decomposed in the circumstances under consideration. Its chlorine unites with the hydrogen of the three offensive compounds referred to, to form hydrochloric (muriatic) acid. The oxygen unites with the nitrogen of the ammonia, and with the phosphorus and sulphur of the others, forming nitric, phosphoric, and sulphuric acids; and the lime combines with these acids, forming nitrate of lime, etc.

Of course it is not maintained that these reactions, and these only, can take place under the supposed circumstances; for the liberated oxygen may, in part, unite with hydrogen, forming water; but the disinfectant results would not be thereby compromised.

When the offensive odor is in the cavity of a tooth, or in the antrum, the *hypochlorite of soda* (liquid chloride of soda) is more convenient than chloride of lime, as it is already in the liquid state. This is also a more pleasant application to the tonsils or fauces, and is better taken internally than the chloride of lime. When used internally, or as a gargle, it should be diluted with pure water.

From the remarks already made, the careful reader will have but little difficulty in *managing* any smell with which his office is likely to be polluted, or himself annoyed. He should never *give* occasion for attention to the odors of tobacco, garlic or onions. If he can not totally govern his appetite in these directions, he should control them as long as possible, then close his office, lay in a bountiful supply of the bulbous roots, have himself arrested, and duly imprisoned in a tobacco warehouse, and there riot in the luxuries of his favorite stinks, while men more civilized wait on his patients.

A fetid perspiration is often a very serious annoyance to persons cleanly in their habits and attentive to their persons. To such we would hint that as the sweat usually gives an acid reaction, alkaline washes are suggested. The butyric acid of the perspiration is readily neutralized in this manner; and the carbonate of potash is as effective and convenient, for thus washing as any other alkaline substance. But the offensive perspiration of the armpits, groins, and feet, is often alkaline; in which case acids would be suggested. The feet are often very difficult to manage in this respect. Some have succeeded admirably by slightly moistening their hose with diluted acetic acid, of course not neglecting the most scrupulous cleanliness. But, in extreme cases, the chloride of lime is the best resort. If the perspiration is acid, the lime is there to neutralize it. And whether acid or alkaline, but few odorous compounds are eliminated, either by secretion, excretion, or putrefaction, that oxygen and chlorine, in their nascent state, can not decompose.

We intend this article to be practical. Many dentists, as well as others, are offensively odorous, either naturally or by art, without being conscious of it. Even the foul-mouthed tobacco-user, with the loathsome narcotic exhaling from every pore, regards his presence as not unpleasant. It is probable that the skunk, too, is blest with a like innocence; for we never hear of its forsaking its den on account of its own odor.

